# Mitochondrial Impairment in Sarcopenia

**DOI:** 10.3390/biology10010031

**Published:** 2021-01-06

**Authors:** Francesco Bellanti, Aurelio Lo Buglio, Gianluigi Vendemiale

**Affiliations:** Department of Medical and Surgical Sciences, University of Foggia, viale Pinto 1, 71122 Foggia, Italy; aurelio.lobuglio@unifg.it (A.L.B.); gianluigi.vendemiale@unifg.it (G.V.)

**Keywords:** protein homeostasis, aging skeletal muscle, mitochondrial dysfunction

## Abstract

**Simple Summary:**

With old age, the strength and size of our muscles worsens with time, affecting our ability to carry out daily activities. Muscle wasting may be more dangerous for some people, causing falls, inactivity, and a loss of self-sufficiency. This severe muscle-wasting condition is called sarcopenia. Mitochondria are sub-cellular organelles involved in the regulation of determinant functions in muscles, such as energy production and programmed cell death. The present review describes muscle modifications and mitochondria alterations occurring in old age, digesting the most important discoveries on mitochondrial changes in sarcopenia. Our comprehensive summary of scientific advances in this field during the last years will be of help for researchers to design future investigations which clarify further aspects of mitochondrial involvement in sarcopenia and define the impact of mitochondria-targeted therapies for the prevention and treatment of sarcopenia.

**Abstract:**

Sarcopenia is defined by the age-related loss of skeletal muscle quality, which relies on mitochondrial homeostasis. During aging, several mitochondrial features such as bioenergetics, dynamics, biogenesis, and selective autophagy (mitophagy) are altered and impinge on protein homeostasis, resulting in loss of muscle mass and function. Thus, mitochondrial dysfunction contributes significantly to the complex pathogenesis of sarcopenia, and mitochondria are indicated as potential targets to prevent and treat this age-related condition. After a concise presentation of the age-related modifications in skeletal muscle quality and mitochondrial homeostasis, the present review summarizes the most relevant findings related to mitochondrial alterations in sarcopenia.

## 1. Introduction

The term “sarcopenia” is derived from the combination of two Greek words, sark (flesh) and penia (loss), and defines the age-related loss of muscle mass and function [1]. Changes underlying sarcopenia include both structural and molecular modifications that alter muscle quality and lead to functional impairment. Other than limitations in muscle mass/quality and function, sarcopenia is associated with several comorbidities including a higher risk of falls and an increased prevalence of metabolic diseases (e.g., obesity and type 2 diabetes mellitus) [2]. Since the percentage of aged people is expected to increase in the next decades, the characterization of specific molecular mechanisms underpinning sarcopenia represents a field of active research to identify therapeutic targets for the prevention and treatment of this healthcare problem.

The pathophysiology of sarcopenia includes impairment in myofiber metabolism and the alteration of muscle satellite cells, causing defective myogenesis with consequent loss of skeletal muscle homeostasis [3]. Sarcopenia is also associated with neurological deficiencies, involving motor neurons and neuromuscular junctions, whose impaired remodeling leads to denervated muscle fibers, contributing to the loss in muscle quality and strength [4]. Furthermore, impaired muscle quality of old age is accompanied by chronic low-grade inflammation, defective anabolic signaling mediated by the growth hormone (GH)/insulin growth factor-1 (IGF-1) pathway, reduced protein intake, and vitamin D insufficiency [5,6,7]. All these findings are linked with the disruption of muscle bioenergetics, which mostly depend on mitochondrial homeostasis and metabolism [8]. Indeed, several investigations indicate that alterations in mitochondrial morphology, function, dynamics, and biogenesis may be the central feature of altered muscle quality and function. After a brief presentation of age-related mitochondrial modifications in skeletal muscle, the present review presents the latest evidence of the role played by mitochondria in the modulation of muscle metabolism in sarcopenia.

## 2. Aging and Skeletal Muscle Quality

Aging is characterized by a loss of about 30% in skeletal muscle mass and 20% in muscle cross-sectional area (CSA) [9]. The rate of skeletal muscle mass loss in 75-year-old subjects is 0.64–0.70% per year in women and 0.80–0.98% per year in men [10]. The prevalence of sarcopenia in 70-year-old subjects is 5–13%, while it is 11–50% in people >80 years old [11]. The European Working Group on Sarcopenia in Older People 2 indicates low muscle strength as the key clinical factor of sarcopenia, applying muscle mass and quality to validate the diagnosis and physical performance to characterize severity [11]. Even though most studies pointed out the loss of skeletal muscle mass as *primum movens* of sarcopenia (resulting in a reduction of muscle strength and performance), changes in strength and performance occur several years before mass loss in older people without major morbidity [10]. Thus, skeletal muscle strength and performance may not only rely on mass, and it is conceivable that, in addition to a reduction in quantity [12], a decline in skeletal muscle quality occurs with age [13]. Skeletal muscle quality is defined as strength (or power) per unit of mass and relies on the architecture and metabolism of muscle tissue [10,14].

### 2.1. Age and Skeletal Muscle Architecture

**Figure 1:**
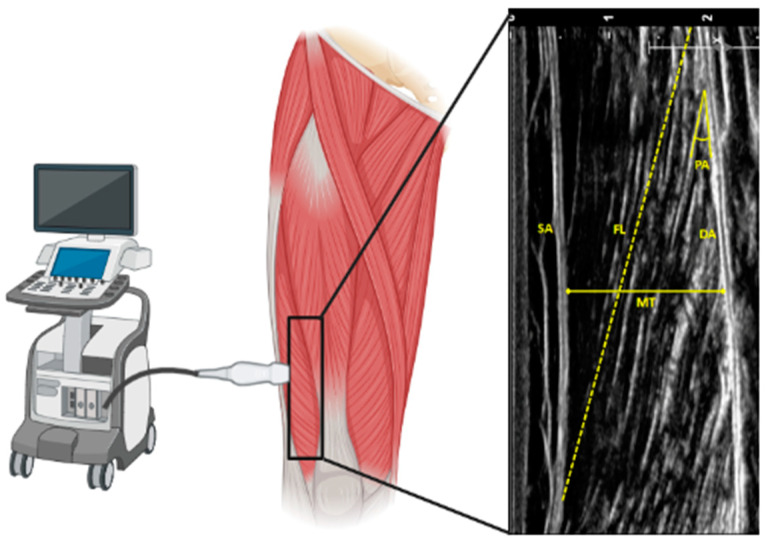
Schematic representation of ultrasound measurement of the architecture of vastus lateralis muscle. SA: superficial aponeurosis; DA: deep aponeurosis; MT: muscle thickness; FL: fascicle length; PA: pennation angle.

Changes in skeletal muscle architecture involve modifications in muscle structure and fiber number, size, and composition. Features of skeletal muscle architecture include CSA, fascicle length (FL, the angle between fascicle and deep aponeurosis), and pennation angle (PA, the length of the fascicle across the deep and superficial aponeurosis, Figure 1). FL affects muscle shortening velocity, while PA is mainly involved in muscle strength [15]. CSA, FL, and PA tend to progressively reduce with age. Studies comparing young and old subjects measured both FL and PA in several skeletal muscles by muscle ultrasonography, which is the most cost-effective method but presents with inter-rater reliability [16]. Even though no reference values are available, FL and PA are lower in 70–81-year-old compared to 27–42-year-old people [14]. Furthermore, old people show a decrease in whole-muscle CSA (19.9%), FL (10.2%), and PA (13.2%) of the gastrocnemius medialis muscle [17]. These changes impact muscle power, defined as the product of force generated and movement velocity. Considering the decrease of muscle function in old age, an average 72% power decline is more noticeable than strength loss in aged (69–82 years) versus adult (19–35 years) subjects [18] since the velocity of shortening will be reflected on myosin heavy-chain ATPase.

Skeletal muscle mass reduction in old age is related to the decrease in the size and number of single muscle fibers [19]. Muscles are classified as fast (appearing morphologically white) and slow (appearing red, because of higher myoglobin content and capillarization, which confer greater oxidative capacity). The histological demonstration of a relationship between the speed of muscle shortening and myosin ATPase activity led to the classification of muscle fibers into type I (slow) and type II (fast) [20]. These modifications rely on the age-induced loss of motor units (MU). A MU is defined by the soma of an alpha motor neuron sited in the ventral horn of the spinal cord and the muscle fibers it innervates. Loss of a MU causes fiber denervation and contributes to muscle atrophy. This loss may be counterbalanced by MU remodeling, characterized by the reinnervation of denervated fibers by neighboring axons, which is defective in sarcopenia [21]. While the composition of vastus lateralis muscle is approximately 70% muscle fibers in adult men, it is reduced to about 50% in old, with an increase in lipid infiltration (steatosis) and connective tissue (fibrosis) [19]. Indeed, fat progressively tends to accumulate below the fascia and within the aged muscle (intermuscular adipose tissue, IMAT). Other than being an independent predictor of gait-speed decline in the elderly, IMAT may impair muscle strength and metabolism [22,23]. On the other side, age-dependent deposition of fibrotic tissue in skeletal muscle is probably dependent on altered regenerative potential [24]. Muscle fibrosis is the final outcome of several events, including recurrent microtrauma, inflammatory cell infiltration, tissue degeneration, and fibroblast proliferation [25]. Finally, the size of skeletal muscle fibers shrinks with age; even though this occurs preferentially in type II fibers, studies also indicate a reduction in the diameter of type I fibers [25,26,27].

### 2.2. Age and Skeletal Muscle Metabolism

Skeletal muscle is the main metabolic tissue in the human body, with a significant consumption of macronutrients and oxygen to produce ATP for contraction. Indeed, skeletal muscle tissue accounts for 60% of total body oxygen consumption in intense exercise. On the other side, skeletal muscle cells dispose consistently of glycogen and phosphocreatine to ensure limited energy in anaerobiosis [28].

Age-dependent decline of skeletal muscle quality is related to the reprogramming of tissue metabolism leading to impaired glucose, fat and protein uptake and utilization, and finally energy production [29]. Alterations in skeletal muscle metabolism in aging present with differences between males and females and are affected by sex hormones [30]. With age, skeletal muscle loss and increase in visceral adipose tissue are higher in males, while females present with reduced capillarization of type II glycolytic myofibers [23]. Macronutrient metabolism in aged skeletal muscle is influenced by the fiber composition of the muscle. Indeed, type I slow-twitch fibers preferably metabolize fatty acids and are characterized by oxidation, while type II fast-twitch fibers rather metabolize glucose anaerobically. As stated before, aging is characterized by a higher loss of type II rather than type I fibers [31]. A reduction in capillarization is also described in old skeletal muscle, with lower nutrient delivery to muscle cells [32,33]. Current evidence supports the close link between the capillary-to-fiber ratio and muscle fiber size, particularly in type II muscle fibers, which are more susceptible to aging-related impairment [34].

Proteins involved in glycolysis and glycogen metabolism, as well as GLUT4 protein (the transporter mediating insulin-dependent glucose uptake), are downregulated [35,36]. Moreover, several age-related alterations in skeletal muscle insulin signaling were reported, contributing to systemic insulin resistance and impaired glucose metabolism [37]. Skeletal muscle lipid accumulation in old age is mostly dependent on alterations in uptake and oxidation. Observations on aged rodents suggest that triglyceride storage in skeletal muscle cells is associated with reduced palmitate oxidation and increased uptake [38,39]. Old people show alterations in protein homeostasis (or proteostasis), characterized by the unbalance between protein synthesis, folding, breakdown, and trafficking, in favor of greater catabolism that can promote loss of skeletal muscle quantity and quality [25]. However, several different factors can influence protein turnover, such as nutritional status, insulin sensitivity, and physical activity [40,41,42]. Alterations related to these factors may partly explain the progressive aging-related decline of muscle quality [43]. Aged skeletal muscle is less receptive to amino acid availability, reducing the ability to promote protein synthesis to counteract breakdown: this concept is defined as anabolic resistance [44]. A classical hallmark of proteostasis disruption is the accumulation of aggregated proteins, which may be promoted by the age-dependent increase in products of oxidative damage [45,46]. In addition, proteostasis loss is conditioned by dysregulation in the ubiquitin-proteasomal and the autophagy-lysosomal systems, the two most important pathways for protein degradation [47].

## 3. Skeletal Muscle Mitochondria Homeostasis

**Figure 2:**
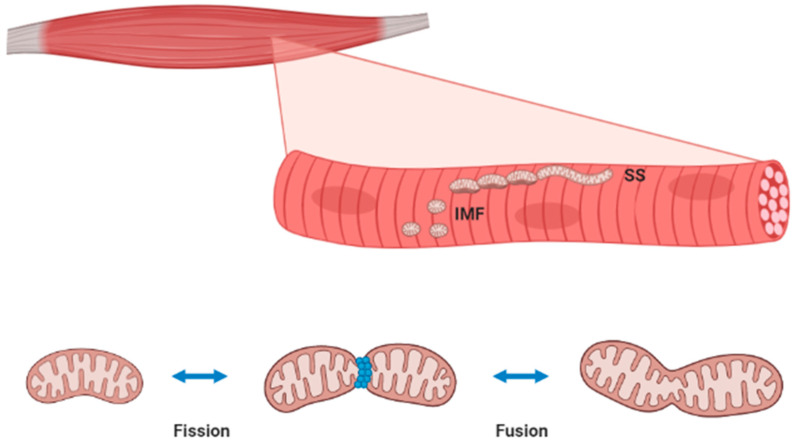
Localization and morphology of subsarcolemmal (SS) and intermyofibrillar (IMF) mitochondria in skeletal muscle (**top panel**). These two distinct mitochondrial subpopulations exist as an uninterrupted reticulum undergoing continuous fission and fusion events (**low panel**). Representing more than 10% of skeletal muscle volume, mitochondria are vital for energy production but also fiber maintenance. A comprehensive discussion on mitochondria in skeletal muscle includes organelle function, dynamics (fission/fusion), biogenesis, and degradation through targeted autophagy (mitophagy). Mitochondrial function is mainly dedicated to ATP production through oxidative phosphorylation (OXPHOS), even though these organelles are also involved in apoptosis, calcium homeostasis, and the production of reactive oxygen and nitrogen species (ROS and RNS, respectively). The biogenesis of ATP in skeletal muscle mitochondria is driven by cytosolic ADP as a product of ATP-consuming reactions. In basal conditions, since ATP needs are low, substrate metabolism and OXPHOS are minimal, but the high-proton motive force leads to a certain production of ROS/RNS [53]. During skeletal muscle contraction, myosin ATPase activity increases ADP bioavailability with consequent utilization of the electrochemical gradient for ATP synthesis and induction of OXPHOS, reducing ROS/RNS generation [54]. ATP production for skeletal muscle contraction mainly occurs in IMF mitochondria, while SS mitochondria mostly provide ATP for active membrane transport and gene transcription [55]. Indeed, the amount of available ATP is determinant to skeletal muscle cell maintenance and proteostasis.

Aerobic capacity, described as the maximum capability of using oxygen to meet the energy needs both at rest and during exercise, tends to decrease with age. Changes in skeletal muscle energy metabolism occur in aged people [14]. Efficient skeletal muscle bioenergetics hinge on mitochondria, and mitochondrial dysfunction is recognized as a major hallmark of aging [48]. Skeletal muscle mitochondria can be localized below the sarcolemma (subsarcolemmal, SS) or between the myofibrils (intermyofibrillar, IMF); SS and IMF mitochondria show different biochemical and morphological features, with different adaptation capacities to exercise and disease (Figure 2) [49,50,51]. A third mitochondrial subpopulation, named perinuclear, was described in skeletal muscle as a continuation of SS around the nucleus, even though it is less characterized than SS and IMF [52].

Mitochondria may initiate apoptosis by the opening of the permeability transition pore (mPTP), causing organelle swelling and the release of cytochrome c and other proapoptotic factors [56]. Opening of mPTP is triggered by ROS/RNS, mitochondrial depolarization, and increased Ca^2+^ concentration. The role of mitochondria in the regulation of Ca^2+^ levels in skeletal muscle is tightly coupled with sarcoplasmic reticulum and cytoplasm crosstalk, modulating not only contraction but also metabolism and intracellular signaling [57,58]. Furthermore, Ca^2+^ excess within mitochondria may trigger ROS/RNS production [59]. Of note, Ca^2+^ uptake capacity is higher in SS rather than IMF mitochondria [60].

Mitochondrial dynamics are related to the ability of these organelles to quickly modulate their size, shape, and distribution by fission and fusion events (Figure 2). To facilitate energy distribution, IMF mitochondria in skeletal muscle are organized in a connected reticulum; on the contrary, SS mitochondria are described as isolated spherical units [51]. Reticulum formation is the product of mitochondrial fusion, leading to the development of a tubular network. On the other side, a mitochondrial reticulum may be fragmented into single organelles by the fission process. While fusion leads to the sharing and rearrangement of macromolecules and attenuation of damages within the network, fission isolates dysfunctional elements from the network to allow their removal [61,62]. Mitochondrial fusion is regulated by proteins such as mitofusins 1 and 2 (Mfn1 and 2), which attach contiguous mitochondrial outer membranes, and optical atrophy 1 and 2 (OPA1 and 2), which mediate blending of mitochondrial inner membranes [63]. On the contrary, mitochondrial fission is modulated by the dynamin-related protein 1 (Drp1), the mitochondrial fission factor (Mff), and fission protein 1 (Fis1), which cooperate to promote organelle separation [64]. In skeletal muscle, type I fibers present with more tubular networks with a higher fusion rate than type II fibers [65,66]. Of note, training exercise promotes mitochondrial fusion in skeletal muscle, leading to increased expression of fusion proteins Mfn1/2 and Opa 1 as well as decreased fission protein Drp1 [67,68]. Even though these changes are mostly described in IFM rather than SS mitochondria, both subpopulations present with a similar adaptive dynamic response to skeletal muscle use [69].

Mitochondrial turnover is balanced by organelle biogenesis and mitophagy. In a steady-state condition, mitophagy is counteracted by an equal rate of biogenesis to preserve mitochondrial content. Changes to this turnover in skeletal muscle are noticeable and related to variations in energy demand. Indeed, to maintain a healthy mitochondrial pool, energy-requiring conditions in skeletal muscle (i.e., exercise) trigger a signaling network that leads to increased biogenesis but also activates mitophagy [70]. Mitochondrial biogenesis involves a synchronized activity on both nuclear (~1000 genes) and mitochondrial (13 critical genes) genomes orchestrated by the peroxisome proliferator-activated receptor-γ coactivator-1α and β (PGC-1α and β), mostly expressed in slow-twitch muscle fibers [71]. PGC-1α activates several downstream transcription regulators such as nuclear respiratory factors 1 and 2 (NRF1 and 2) and mitochondrial transcription factor A (Tfam), with consequent expression in the cytosol of proteins that are transported within mitochondria through the protein import machinery (PIM) [72]. Of interest, exercise promotes mitochondrial biogenesis in skeletal muscle via PGC-1α activation [73]. Indeed, muscle contraction increases the concentration of AMP altering the AMP/ATP ratio, with consequent stimulation of AMP-activated protein kinase (AMPK), which phosphorylates PGC-1α leading to increased mitochondrial biogenesis [74]. Both muscle contraction and AMPK may increase the level of the oxidized form of nicotinamide adenine dinucleotide (NAD^+^), which stimulates the deacetylation activity of silent mating type information regulator 2 homolog 1 (SIRT1), activating PGC-1α [75]. The increase in the AMP/ATP ratio and resulting AMPK activation also initiates mitophagy through phosphorylation of Unc-51-like autophagy activating kinase (ULK1), the most described upstream mitophagy protein [76]. Furthermore, mitophagy can be promoted by the loss of mitochondrial membrane potential, which leads to the accumulation of phosphatase and tensin homolog-induced putative kinase protein 1 (PINK1), with consequent phosphorylation of PARKIN and enhanced mitochondrial ubiquitination [77]. Finally, mitophagy can be triggered by endogenous mitochondrial membrane-bound receptor proteins, such as BCL2/adenovirus E1B 19kD interacting protein 3 (BNIP3), which activates the autophagosome [78]. Of note, slow-twitch fibers and endurance exercise-trained skeletal muscles present with increased levels of BNIP3 protein [79]. This evidence supports the key role of mitophagy in the control of mitochondria quality in skeletal muscle.

## 4. Age-Related Mitochondrial Alterations and Sarcopenia

Mitochondrial dysfunction is determinant in age-related loss of skeletal muscle mass and strength. Indeed, protecting mitochondria is a determinant to preserve proteostasis in skeletal muscle. To date, a growing body of evidence on mitochondrial impairment in sarcopenia has been provided by both animal and human studies (summarized in Table 1).

Dysfunctional mitochondria are associated with both ATP depletion and ROS/RNS excess, with the consequent activation of harmful cellular pathways. A decrease in mitochondrial mass, activity of tricarboxylic acid cycle enzymes, as well as O_2_ consumption and ATP synthesis occurs in aged skeletal muscle tissue [107]. Changes in function, dynamics, and biogenesis/mitophagy could explain in part alteration in oxidative capacity and content of skeletal muscle mitochondria. Furthermore, mitochondrial dysfunction induces the activation of apoptosis, potentially impairing skeletal muscle quality [108].

Several mitochondrial functions are impaired in old in comparison to young skeletal muscle, including the activity of metabolic enzymes and oxidative phosphorylation (OXPHOS) complexes (i.e., citrate synthase and cytochrome c oxidase), respiration, protein synthesis, and ATP production rate (mostly dependent on an increase in mitochondrial uncoupling) [56,80,95,96,109]. Nevertheless, the intensity and duration of physical activity may be determinants for the preservation of mitochondrial function in old skeletal muscle [89,97,110]. Comparison analysis of transcriptome between young and old skeletal muscle from animal models and humans shows a decrease in mitochondrial gene expression as an effect of age, even though proteomic studies concluded with controversial results, indicating the need for further research (reviewed in [111]). It is worth to note that genes related to mitochondrial structure and function are downregulated in older women compared to men, indicating that females may be more predisposed to skeletal muscle impairment with age [98].

The reduced mitochondrial content in aged skeletal muscle may be also related to lower PGC-1α gene and protein expression, which is reported both in slow- and in fast-twitch fibers [106,109,112]. However, the molecular mechanisms that underpin this reduction are worth further investigation. Apart from PGC-1α, different studies show divergent results in the levels of its downstream transcription factor Tfam in old skeletal muscle [81,87,100]. Changes related to mitochondrial content and function in old skeletal muscle may also be related to a reduced amount, increased mutations, deletions, and rearrangements of mitochondrial DNA (mtDNA) [101,102]. The protein level of skeletal muscle PIM is similar between young and old animals, suggesting that molecular chaperones and translocases are not involved in the mitochondrial impairment of aged skeletal muscle [90,91]. Alterations in mitochondrial electron transport chain (ETC) and mtDNA, sustained by the oxidative damage, occur in sarcopenia [92]. Furthermore, a greater prevalence of mtDNA deletion mutations is described in skeletal muscle fibers, which were more subjected to oxidative damage [113,114]. An age-dependent increase in skeletal muscle fibers presenting with alterations of mitochondrial enzymes due to mtDNA deletion mutations is reported both in rhesus monkeys presenting with early-stage sarcopenia and in humans [94,103]. The sedentary lifestyle of aging is associated with mitochondrial dysfunction and oxidative damage in human skeletal muscle, but physical activity in old age may prevent mitochondrial-dependent sarcopenia [104,115]. In a transgenic mouse model, mtDNA mutations were determinant for ETC assembly and function, with consequent impaired mitochondrial bioenergetics and loss of ATP homeostasis, enhancing skeletal muscle apoptosis and sarcopenia [116]. Defective mitochondrial ETC was also described in spinal motor neurons from older humans, contributing to fiber denervation and loss of skeletal muscle quality [117]. Of note, denervation of single skeletal muscle fibers induces the overproduction of mitochondrial ROS/RNS even in neighboring innervated fibers, suggesting a complementary mechanism in the pathogenesis of sarcopenia [118].

Morphological studies in aged skeletal muscle show giant mitochondria with disrupted cristae [112]. Moreover, SS mitochondria appear fragmented and disposed in a thin layer, while IMF mitochondria are less reticular compared with young muscle [67]. Of note, a reduction in IMF size was described in older adults; this was particularly evident in women rather than men, even though sex did not affect the difference in whole muscle size [105]. Altered morphology in old skeletal muscle mitochondria may be the consequence of impaired mitochondrial dynamics, with a disbalance in favor of fission rather than fusion [106]. Mutations in mtDNA may lead to dysregulation of mitochondrial dynamics in sarcopenia, as suggested by results from old mice expressing a defective mtDNA polymerase gamma, which showed higher mitochondrial fission in skeletal muscle [119]. However, a comparison between young versus old mice revealed a higher mitochondrial fusion index (Mfn2-to-Drp1 ratio) in aged skeletal muscle [85]. A shift toward mitochondrial fusion rather than fission was also reported in skeletal muscle of very old hip-fractured patients [120]. A knock-out of fusion-related Mfn1/2 in skeletal muscle showed higher mtDNA mutations and tissue atrophy [121]. Nevertheless, skeletal muscle atrophy and degeneration were also reported from the genetic deletion of fission-related Drp1 [122]. Thus, the actual changes of mitochondrial dynamics in skeletal muscle and their involvement in sarcopenia need to be clarified as well as the potential impact of age-associated alteration in mitochondrial dynamics of motor neurons.

Impaired mitochondrial biogenesis is crucial to determine the loss of skeletal muscle quality and sarcopenia. Both mitochondrial homeostasis and OXPHOS in skeletal muscle are regulated by PGC-1α, the master regulator of mitochondrial biogenesis, which is stimulated by contractile activity and induces fiber-type switching from glycolytic toward oxidative fibers [123]. Nevertheless, age-related reduction in mitochondrial biogenesis may be supported by the impaired response of PGC-1α to exercise training [124]. The potential role of PGC-1α-induced mitochondrial biogenesis as a therapeutic target for sarcopenia is suggested by several preclinical studies, which used agents such as ghrelin, trimetazidine, exerkine, and 5,7-dimethoxyflavone to reverse sarcopenia [125,126,127,128]. Such positive effects on metabolism and proteostasis pave the way for future clinical trials. PGC-1α overexpression in skeletal muscle inhibits mitophagy, which is enhanced during aging [82]. A cross-sectional study performed in physically inactive frail older women described the downregulation of genes related to mitophagy [129].

The reduced capacity of skeletal muscle cells to remove damaged organelles could be another cause of mitochondrial alteration in aging. Studies performed on rodent models describe controversial results on mitophagy modulators in aged skeletal muscle [82,103,128]. A further investigation reported data indicative of increased mitophagy but lysosomal dysfunction in skeletal muscle from old mice, suggesting that lysosomal dysfunction may cause accumulation of disrupted mitochondria [130]. Nevertheless, further investigation on the role of mitophagy in old skeletal muscle is needed in humans. Mitophagy and its related modulatory proteins are enhanced in rodent models of sarcopenia [84,93]. The deletion of the mitofusin 2 gene in skeletal muscle impairs autophagy and activates an adaptive mitochondrial quality control pathway in mice [131]. A mechanism complementary to mitophagy includes the delivery of mtDNA and mitochondrial components through extracellular vesicles (EVs), named mitochondrial-derived vesicles (MEVs) [132]. Of note, older adults affected by physical frailty and sarcopenia presented with higher circulating EVs with respect to age-matched controls, but mitochondrial components were lower, suggesting an alteration in the trafficking of MEVs in old skeletal muscle [133]. Divergent reports on mitophagy in sarcopenia suggest further investigations on this topic since this aspect could be an interesting therapeutic target. Indeed, the overexpression of the mitophagy regulator Parkin in mouse skeletal muscle attenuates sarcopenia by increasing mitochondrial content and enzymatic activities [134].

## 5. Age-Related Apoptosis and Sarcopenia

Apoptosis is a determinant for skeletal muscle homeostasis in adults. This process is triggered by signals, which include ROS and RNS, death receptor ligands, calcium deregulation, and alterations in B-cell lymphoma (Bcl)-2 family proteins, followed by a cascade of cytosolic protein-cleaving enzymes called caspases, with consequent DNA fragmentation [135]. Apoptosis can be activated by extrinsic (or ligand-induced) and intrinsic pathways. An example of extrinsic apoptosis involves the TNF receptor (TNFR) superfamily, with the consequent activation of caspases. The intrinsic pathway may be mitochondrial-dependent or mitochondrial-independent. The first pathway may involve the mitochondrial release of cytochrome c, which aggregates with caspase-9, apoptosis protease activator protein (Apaf)-1, and dATP in the cytoplasm, forming an apoptosome, which in turn activates caspase-3, leading to apoptosis [136]. The second pathway involves caspase-12, which is triggered by perturbations of intracellular calcium homeostasis and in turn activates caspase-9 and -3, independent of cytochrome c release [137]. Apoptosis may be also mediated by caspase-independent mechanisms, characterized by the mitochondrial release of apoptosis-inducing factor (AIF) and endonuclease G (EndoG), followed by DNA fragmentation [138].

Aging is associated with increased apoptosis of rodent skeletal muscle, characterized by mono- and oligonucleosome fragmentation [139]. Apoptosis in aging skeletal muscle and sarcopenia may be sustained by both mitochondria-independent and mitochondria-dependent pathways. Involvement of the mitochondria-independent pathway is provided by evidence that TNF-α may trigger apoptosis in type II with respect to type I skeletal muscle fibers. TNF-α-induced apoptosis is linked to reduced muscle mass, cross-sectional area, and fiber number [140]. Mitochondria-dependent apoptosis was also investigated in old skeletal muscle. Indeed, with respect to young muscle, mitochondria from aged skeletal muscle showed a higher ROS/RNS production rate and lower Ca^2+^ internalization, with consequent mPTP opening, cytochrome c release, and DNA fragmentation, markers of myocellular apoptosis [56,141]. Of note, training exercise is able to decrease the mitochondrial delivery of proapoptotic proteins and the consequent DNA fragmentation [142,143]. In old skeletal muscle, mitochondrial dysfunction triggers a caspase-independent apoptotic pathway that supports the loss of muscle quality [62]. Several investigations indicate the involvement of mitochondrial-dependent apoptosis in rodent models of sarcopenia [53,97,139,140]. Furthermore, apoptotic signaling in skeletal muscle is associated with impaired muscle mass and performance in older people [63]. Ca^2+^ retention capacity is lower in skeletal muscle mitochondria of old with respect to young men, suggesting mPTP sensitization to apoptosis [141]. Apoptosis driven by mitochondrial dysfunction in skeletal muscle cells represents a further therapeutic target to counteract sarcopenia, as indicated by both in vitro and ex vivo investigations [144,145].

## 6. Conclusions

**Figure 3:**
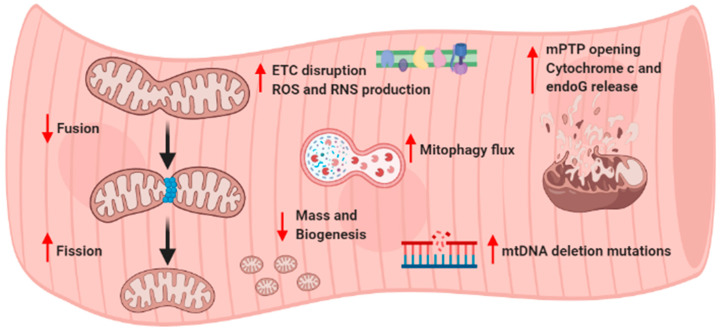
General summary of altered skeletal muscle mitochondrial pathways in sarcopenia. ETC, electron transport chain; ROS, reactive oxygen species; RNS, reactive nitrogen species; mPTP, mitochondrial permeability transition pore; endoG, endonuclease G; mtDNA, mitochondrial DNA.

Mitochondrial disruption in skeletal muscle is the main event in the pathogenesis of sarcopenia (Figure 3). Knowledge of several pathways controlling mitochondrial function, dynamics, biogenesis/mitophagy, and apoptosis, as well as their impact on skeletal muscle quality in aging, has significantly progressed during the last decades. Nevertheless, several aspects need to be clarified in sarcopenia, such as the crosstalk between motor neuron and skeletal muscle mitochondria, the effective role of mitochondrial dynamics and mitophagy in the regulation of proteostasis, and the interplay and adaptation of the components constituting the mitochondrial quality control system. Further investigation is required to describe the impact of nutritional and pharmacological mitochondria-targeted interventions together with exercise programs, leading to the definition of clinical practice guidelines for the prevention and treatment of sarcopenia.

## Figures and Tables

**Table 1:** A list of studies providing evidence of mitochondrial alterations between young and old skeletal muscle from animals or humans. TFAM, mitochondrial transcription factor A; mtDNA, mitochondrial DNA; PGC-1α, peroxisome proliferator-activated receptor-γ coactivator-1α; ROS, reactive oxygen species; OXPHOS, oxidative phosphorylation; CS, citrate synthase; mPTP, mitochondrial permeability transition pore; ETC, electron transport chain.

Species	Age Groups Studied	Relevant Finding in Old Skeletal Muscle	Reference
C57Bl/6 Mice	7 vs. 30 months old	↓ mitochondrial coupling	[80]
C57Bl/6 Mice	2–15 months old	↑ TFAM and mtDNA content	[81]
C57Bl/6 Mice	2 vs. 24 months old	↑ mitophagy ↑ mitochondrial fission	[82]
C57Bl/6 Mice	11–13 vs. 25–27 months old	↓ autophagy	[83]
C5l/6 Mice	3 vs. 18 months old	↓ mitochondrial content ↓ mitochondrial biogenesis ↑ mitophagy flux	[84]
Mice	8–12 vs. 88–96 weeks old	↑ mitochondrial fusion index	[85]
Wistar Rats	3 vs. 26 months old	↓ mitochondrial mass ↓ PGC-1α protein ↑ Fis1 and Mfs1 proteins	[86]
Wistar Rats	3–28 months old	↑ mtDNA deletions ↓ respiratory enzymes	[87]
Fischer 344 Brown Norway Rats	5 vs. 35 months old	↓ mitochondrial size ↑ mitochondrial fission proteins	[67]
Fischer 344 Brown Norway Rats	6 vs. 24 months old	↓ autophagy	[88]
Fischer 344 Brown Norway Rats	6 vs. 36 months old	↓ mitochondrial content ↑ mitochondrial ROS production ↑ cytochrome c and endonuclease G release	[56]
Fischer 344 Brown Norway Rats	8–10 vs. 35–36 months old	↓ OXPHOS proteins, CS activity, state III respiration, mPTP function ↑ free radical leak	[89]
Fischer 344 Brown Norway Rats	6 vs. 35–38 months old	=mitochondrial protein import machinery	[90]
Fischer 344 Brown Norway Rats	6 vs. 36 months old	↑ protein assembly =protein import	[91]
Fischer 344 Brown Norway Rats	5, 18, 36 months old	↑ ETC abnormalities	[92]
Fischer 344 Brown Norway Rats	5–6 vs. 35–36 months old	↑ mitophagy flux	[93]
Rhesus Monkeys	6, 9, 12 years old	↑ enzyme abnormalities ↑ mtDNA deletion mutations	[94]
Humans	17–91 years old	↓ respiratory activity of complex I, II, and IV	[95]
Humans	18–89 years old	↓ mtDNA, mRNA, and mitochondrial proteins ↓ mitochondrial ATP production	[96]
Humans	29–80 years old	↓ oxidative capacity	[97]
Humans	22–75 years old	↓ mitochondrial transcriptome	[98]
Humans	25–72 years old	↓ ETC proteins ↓ ROS production	[99]
Humans	21–88 years old	↑ TFAM mRNA and protein	[100]
Humans	20–71 years old	↑ mtDNA rearrangements	[101]
Humans	49–93 years old	↑ mtDNA deletion mutations	[102]
Humans	20–80 years old	↑ mtDNA deletions	[103]
Humans	20–75 years old	↓ mitochondrial enzymes activity ↓ mitochondrial biogenesis	[104]
Humans	21–75 years old	↓ IMF mitochondrial size	[105]
Humans	22–82 years old	↓ mitochondrial respiration ↓ PGC-1α, COX I, and OPA proteins ↑ mitochondrial protein import machinery	[106]

## Data Availability

Not applicable.
